# Pediatric burn rehabilitation: Philosophy and strategies

**DOI:** 10.4103/2321-3868.118930

**Published:** 2013-09-18

**Authors:** Shohei Ohgi, Shouzhi Gu

**Affiliations:** 1 School of Rehabilitation Sciences, Seirei Christopher University, Shizuoka, Japan; 2 School of Rehabilitation Sciences, Seirei Christopher University, Shizuoka, 433-8558 3453, Mikatabara, Kita-Ku, Hamamatsu, Japan

**Keywords:** Burns, rehabilitation, pediatric, philosophy

## Abstract

Burn injuries are a huge public health issue for children throughout the world, with the majority occurring in developing countries. Burn injuries can leave a pediatric patient with severely debilitating and deforming contractures, which can lead to significant disability when left untreated. Rehabilitation is an essential and integral part of pediatric burn treatment. The aim of this article was to review the literature on pediatric burn rehabilitation from the Medline, CINAHL, and Web of Science databases. An attempt has been made to present the basic aspects of burn rehabilitation, provide practical information, and discuss the goals and conceptualization of rehabilitation as well as the development of rehabilitation philosophy and strategies.

## Introduction

It has been reported that over half a million children are hospitalized with burn injuries each year throughout the world, with the majority occurring in developing countries. The injuries can lead to severe functional, social, and psychological impairment.[1–3] Pediatric burns differ from adult burns in many aspects. Their skin is more sensitive and less resistant to heat, and because it is harder for them to escape from the burning object, this may lead to longer exposure, which may increase the burn severity. Pediatric patients have a smaller body than adults, with a greater body surface area in relation to their weight. Burns can leave a pediatric patient with severely debilitating and deforming contractures, which can lead to significant disability when left untreated. Therefore, burn rehabilitation is not to be undertaken by individuals, but should involve a multidisciplinary team so that every aspect of the child’s physical, psychological, and social needs is met during hospitalization and following discharge. Complex social issues often impact the delivery of a child’s care and, therefore, require skilled personnel to manage adjustment to hospitalization.[4,5] The rehabilitation of pediatric patients with burn injuries starts from the day of injury, lasts for several years, and requires a multidisciplinary effort. A comprehensive rehabilitation program is essential to decrease the patient’s post-traumatic effects and improve functional independence.[6] Such a difference can be made to the long-term quality of life (QOL) of pediatric burn patients through the dedication of the individuals within the burn team, their commitment to caring for the patient, and their encouragement of patient participation and full engagement in rehabilitation.

**Table 1:** 

Access this article online	
**Quick Response Code:**	**Website:** www.burnstrauma.com
	**DOI:** 10.4103/2321-3868.118930

In this article, we reviewed literature from the Medline, CINAHL, and Web of Science databases. We herewith share the basic aspects of burn rehabilitation, provide practical information, and discuss wider issues such as the goals of rehabilitation, the conceptualization of rehabilitation, and the development of rehabilitation philosophy and strategies.

## Principles of child development and the concept of pediatric burn rehabilitation

**Figure 1:**
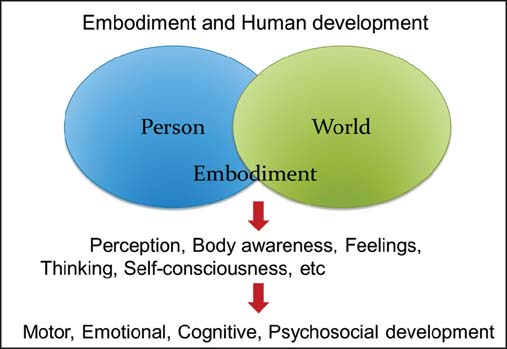
Embodiment as a principle of child development and the concept of pediatric rehabilitation.

Human development and behavior emerge from active engagement with the surrounding world. Embodiment refers to the self-organizational grounding of complex systems in physical experience and interactions. Thus, embodiment is the claim that our perception, body awareness, thinking, feelings, self-consciousness, desires, and motivations, that is to say the way we behave, experience, and live in the world are contextualized by our being active agents with this particular type of body[7] One current theoretical framework for human development views embodiment as the emergent product of motor, emotional, cognitive, and social development. Therefore, pediatric rehabilitation should be focused on embodiment, referring both to the body as a physical structure and its function as well as to the body as a form of lived experience, actively engaged with a world of socio-cultural and physical objects [Figure 1].

To emphasize children’s embodied experiences in pediatric rehabilitation, we have focused on family-centered, relationship-based, activity-focused, and positive feedback interventions as the central principle of the well-being of the child and his/her family. Family-centered and relationship-based interventions are provided to allow the patients to feel like genuine partners of the medical staff in their treatment and rehabilitation process. Rehabilitation should be thoroughly committed to collaborating with children and their families in all aspects of patient care. Within this partnership, the opinions of the patients and their families are recognized and respected. Activity-focused interventions are consistent with the current models of intervention for children with disabilities and handicaps. In these interventions, effective movement experiences are at the heart of active motor learning processes in the acquisition of developmental skills that are important for daily activities. A passive approach typically consists of the interventions directed toward reducing secondary impairments such as joint limitations or soft-tissue contractures associated with muscle tone disorders. However, it is not administered within the context of purposeful activities. Intrinsic motivation is more desirable and results in better functional activity and outcomes; patients are more likely to engage in the task willingly and to work to improve their skills, which will increase their functional activities and capabilities. Previous studies suggest that positive feedback can increase intrinsic motivation in comparison to lack of positive feedback or negative feedback. Additionally, positive feedback is related to self-efficacy and intrinsic motivation. These intervention rules are derived from one of the most important theories of child education developed by Vygotsky, known as the Zone of Proximal Development (ZPD).[8] The ZPD is the area between the “actual developmental level and the potential developmental level through problem solving under adult guidance or minor assistances.” Vygotsky believes that the role of education is to provide children with the experiences that are in their individual ZPD, thereby encouraging and advancing their individual learning. In rehabilitation therapy for individual children with disabilities and handicaps, it is important to assess a child’s individual ZPD and then to intervene within this ZPD. As a result, children solve their developmental problems and then achieve the next developmental stage.

More recently, based on motor control and learning principles, the emphasis has been shifted away from paying attention to what was thought to be the underlying cause of a child’s movement difficulties to models that emphasize the importance of motor learning within the context of performing tasks. Therapeutic approaches focusing on changing the task and the environment rather than a child’s impairments can be a viable treatment strategy. A task-oriented approach is based on models of the motor learning principles of spaced practice and intermittent feedback and is used to facilitate real-world activities. This approach involves the practice of goal-directed, functional movement and is performed in a natural environment. In addition, it involves a variety of practices to help patients derive optimal control strategies for solving motor problems. Such active training in functional tasks within a meaningful environment is known to improve motor control, functional recovery, and strength. Therapeutic activities should vary and enhance the patient’s active participation. The setup of the environment should include all factors that might regulate a specific task practice. Lastly, appropriate feedback should be available to enhance both motor learning and relearning processes.[9]

## A rehabilitation philosophy and strategies for children with burn injuries

It is very important that pediatric burn units realize the need for a child psychiatrist and a psychotherapist in their rehabilitation team. Good psychotherapy along with burns-related treatment will go a long way to enhance the QOL of these patients.[10] To create a success story for children with burn injuries, the most effective health care interventions for the child and family are delivered through a multidisciplinary team care approach. The process of rehabilitation requires efficient communication both between medical and care professionals and the patient and his/her family to effectively address all rehabilitation goals. Psychosocial recovery and social re-integration require a cooperative effort from social members of the society. The International Classification of Functioning (ICF), Disability, and Health[11] model utilizes a common language in the understanding of health and health-related status, including the subject’s biology (body functions and structure) and personal activity and participation (social perspectives), among multidisciplinary teams as well as clinical problem-solving tools in the rehabilitation process. Each professional in the multidisciplinary team has an important function and responsibility in each domain. For example, a physician takes responsibility for the “health condition” (burn treatment), and Physical Therapy (PT)/ Occupational Therapy (OT) takes the central role in the “body functions and structure” and “activities” domains. A nurse may cover all the domains. Thus, the ICF model facilitates the responsibility of each professional and provides comprehensive and coordinative interventions to the child and his/her family. The ICF model is useful in describing patients’ individual goals, taking into consideration the patients’ and families’ needs and wishes, and in optimizing the management of the rehabilitation process.

## Pediatrics burn treatment and rehabilitation

Burn rehabilitation should start at the time of the burn injury to achieve optimal individual functioning, activity, and long-term QOL. Early rehabilitation focused on long-term goals, with a collaborative team approach, is very important to prevent post-acute sequelae, such as scarring and joint contractures, and to achieve early functional recovery. Better rehabilitation goals are achieved using treatments such as pain control, pressure garments, splinting and positioning, exercise, and ongoing mental and psychosocial support and education of the child and his/her family.

## A collaborative multidisciplinary team approach and family support

**Figure 2:**
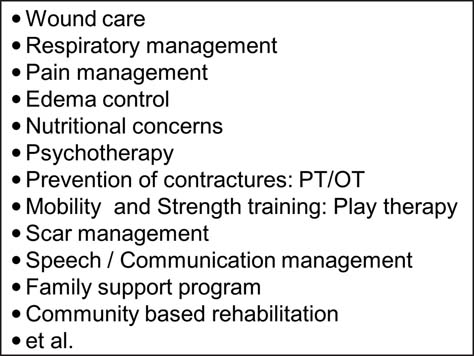
A multidisciplinary team approach for burn rehabilitation.

The most important point is that burn rehabilitation is a multidisciplinary, coordinated team approach with the family at the center. A multidisciplinary team is required in every recovery stage because the treatment and care of a child with a burn injury includes the child’s physical and psychological state as well as complex family and social issues. A multidisciplinary team including doctors, nurses, occupational and physical therapists, social workers, psychiatrists, music therapists, play therapists, teachers, and volunteers is involved in assessing and planning the medical care and rehabilitation process for the child and his/her family [Figure 2]. Comprehensive medical and rehabilitation programs provided by a multidisciplinary care team guarantee the most significant improvements in a child’s physical and psychological functioning as well as family support and his/her social re-integration.

Educating and providing information to the family can prevent misunderstanding about the physical treatment and alleviate stress and anxiety about post-burn psychological and emotional responses.[12,13]

## Minimizing the impairment of bodily functions and structures

According to the ICF model, impairments in body structures and functions are consequences of a disorder or pathological process. Physiologic functions of the body and its structures are lead functional limitation of the child’s daily activities and participation involved in routines of social life. Hypertrophic scarring is common following a burn injury and may cause significant functional and cosmetic impairment. Scar management following a burn injury is a long and often painful process; it is not something that can be carried out for a few weeks and then abandoned, but is something that must continue for many months to minimize post-burn complications. There is no consensus regarding the best treatment to reduce or prevent hypertrophic scarring.[14] However, the prevention of scar tissue contracture and deformity is typically the first step in rehabilitation. While acute care is dependent on the depth and extent of the injury, age of the child, presence of infection, and degree of wound healing, it may involve vascular and respiratory care, nutritional support, inhalation injury treatment, infection, and wound management. Positioning, splinting, and exercise are required for the prevention of contractures or joint stiffness. Positioning is crucial, particularly in the early stage of recovery, and it is effective in (a) decreasing edema by elevation of the extremities and exercise, (b) maintaining range of motion (ROM), (c) preventing contractures and joint stiffness, and (d) preventing muscle shortness. Splinting and/or serial casting are helpful in maintaining positioning and/or increasing the ROM, particularly at night. Active and passive ROM exercise from the onset of injury is very important during all phases of burn wound care in preventing scar contractures and limb deformities and for skin graft preservation and healing and reconstruction. ROM exercise also helps lessen the effect of edema fluid and immobilization, maintain joint mobility and muscle function, and decrease the physical deconditioning resulting from prolonged rest. The use of paraffin, ultrasound, continuous passive motion (CPM), massage, and fluid therapy can both provide pain relief and increase the ROM. Massage has many benefits such as decreasing skin hypersensitivity and edema. It can also be helpful in managing pain, anxiety, and emotional recovery from the burn trauma. Pressure garments are used for the management of hypertrophic scars, which can restrict the ROM and subsequently lead to contractures. Pressure should be applied as early as possible. Early ambulation exercise maintains balance, lowers extremity ROM, and decreases the risk of deep venous thrombosis. As soon as the patient’s condition allows, ambulation should begin. Play therapy is the best strategy for increasing function through activities involved in strengthening, endurance, and motor function and skills. Physical activities, including body movement, balance, and kinesthetic sense through play therapy are related to skeletal muscle strength, cardiorespiratory tolerance, and visceral system activities. Play therapy also has the capacity to address and resolve issues of anxiety, depression, and other emotional issues highly correlated with long-term medical and psychological health. Play interactions between the child and the parents are critical for healthy physical and emotional recovery. Aquatic therapy is a great choice for physical improvement through the properties of water. It enhances cardiorespiratory tolerance and gross motor development and recovery, as it involves most of the large muscle groups of the body while enabling children to exercise in a non-weight-bearing environment. Resistance and aerobic exercise programs have also been shown to improve muscle strength and power and lead to lean body mass gain during rehabilitation.[15]

## Pain control and management (short-term outcomes)

Early commencement of rehabilitation is the key to compliance with treatment and maximizing long-term outcome. Children with burn injuries suffer severe pain at the time of the burn and during treatment and rehabilitation. As pain has adverse physiological and emotional effects and slows down the functional recovery process, pain management is essential to better outcomes during all stages of treatment and rehabilitation.[16,17] The purposeful activity based on playing and games can reduce pain and improve hand movement and function better than rote exercise.[18] Moreover, poor pain management can lead to anxiety, distress, and a lower pain tolerance threshold. Research suggests that procedural pain-associated anxiety and distress may increase over time in burn-injured patients. Anxiety and distress are significantly related to overall pain. Pain management requires a collaborative plan developed by the burn team that includes pharmacological treatment. Pain assessment should lead to physical and psychological pain-relieving interventions focused on physical, emotional, and family factors. Attention should be given to a child’s environmental conditions such as a parent’s emotional state and social conditions. There is also evidence that the use of non-pharmacological treatments can decrease pain and lessen the use of pain medications. Such strategies for pain management include education, distraction, relaxation, cutaneous stimulation, bio-feedback, imagery (virtual reality), and behavioral treatment. Patients often have pain that generally increases with wound care, dressing changes, and rehabilitation therapy (e.g. ROM exercise). Functional therapy and dressing changes require immediate relief through pre-medication or cessation/rest of the activity. Distractions such as music therapy, play therapy, movies, and applied virtual reality may also be useful in helping a child cope with pain.

## Psychosocial management (long-term outcomes)

A burn injury can be a traumatic experience with social, physical, and psychological consequences.[19,20] Most children with burn injuries have anxiety, depression, and oppositional behavior, withdraw from activities, and exhibit adverse effects on body image and self-esteem. Additionally, between 25% and 33% of burn-injured children eventually develop post-traumatic stress disorder (PTSD),[21,22] which represents some of the core features of a child’s reactions to diverse traumatic events. Burn services should include clinical psychology to provide specialist mental health services for the affected child and his/her family. Psychosocial experts (psychologists, psychiatrists, and social workers) can help a multidisciplinary team develop a treatment plan that will facilitate the adjustment of the child and his/her family.[23] Effective psychological assessment and management should be minimized to ongoing psychological problems and mental health difficulties. Assessment and management of emotional distress, pain coping strategies, grief, and other mental health issues and dealing with feelings surrounding their body image are necessary to ensure proper treatment and rehabilitation planning. The most significant psychosocial adjustment factor for burn-injured children appears to be family support. Family members play a critical role in the long-term care and rehabilitation of a burned child. When a child is burned, the whole family is emotionally affected. Most parents appear to experience shock, guilt, and anxiety. It is important to recognize that family members may experience a range of grief and loss reactions. As part of the multidisciplinary team, psychologists, psychiatrists, and social workers provide expertise in successfully assisting children and their families in the management of psychological problems, emotional disorders, and the consequences of injury.

## Follow-up and community-based rehabilitation

Continuing rehabilitation after discharge from a hospital environment is required to ensure the functional recovery and social reintegration of patients and their families. Effective discharge planning can aid functional recovery, and the patients can continue their improvement outside of a hospital setting in a more home-like and social environment. Following treatment and rehabilitation during the hospital stage, physical complications are particularly challenging in pediatric patients. It is well recognized that programs based on exercise therapy can have a significant impact upon children’s physical and psychosocial well-being.

## Rehabilitation assessment

During the rehabilitation process, information about the daily lives of the children and their families should be obtained for different environments, such as the home and school, and family relations; lifestyles, and socioeconomic and cultural factors should also be considered. The most significant limitations of the long-term QOL for burned children do not appear to be functional impairment, but rather physiological issues and social participation. The disfigurement, unhappiness with their appearance, social anxiety, maladaptive coping, and social discomfort are more likely to influence long-term QOL, compared to the physical results of severe burn injuries.[24] Monitoring and assessment of PTSD symptoms (hyperalertness, nightmares, chronic fearfulness) and early care are needed for appropriate treatment.

Burned children reported lowered QOL, particularly related to scarring and appearance; however, they reported normative self-concept. This may be because of self-concept being a psychological trait, whereas Health Related Quality of Life (HRQoL) is influenced by societal norms and expectations. Psychosocial support is necessary to build positive coping strategies and manage the unpleasant social experiences that may reduce QOL.[25] Long-term emotional outcomes depend upon the family’s psychological care for a child with burn injuries. Parents are extremely important, as they generally have the greatest influence on the child’s life. Therefore, parental psychosocial well-being and parenting skills must be a primary concern in the treatment and rehabilitation process for burned children. Early and adequate care for the family is essential from the onset. Family dysfunction is often evident in the 2–3 months after returning home with a burned child and can later on became a potential marker of psychosocial need.[26] A patient’s need for psychosocial intervention may occur later in their care. Education is required from prevention to rehabilitation, and education in the community, child health centers, schools, and social groups can be used to target parents. Phillips *et al.*, suggested that the key elements for psychosocial support following burn injury in a family support program are (a) normalizing the family member’s reactions to the burn; (b) advice, support, and information regarding scar permanence, realistic outcome expectations, acceptance of altered appearance, and potential aftereffects of the burn; (c) support in understanding how a burned individual may change or respond following injury and advice regarding constructive methods of coping with altered family dynamics and aftereffects of burns; and (d) advice to enable family members and their burned relatives to effectively address potentially uncomfortable social encounters.[27]

**Figure 3:**
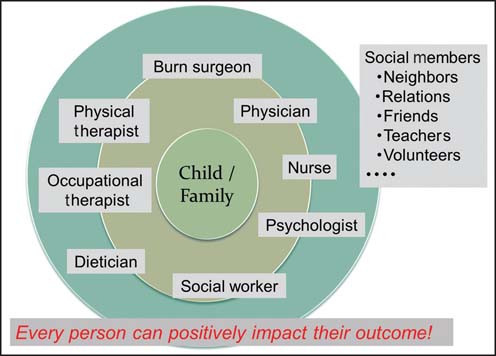
Burn treatment and rehabilitation.

Return to normal daily activities, such as attending school, should be encouraged and supported by return-to-school visiting programs[28] and burn campaign[29] programs. Therapeutic and educational group work programs for the child and his/her family are important to facilitate psychosocial adjustment and promote a positive community understanding of their disability/handicaps. Supportive groups for burn survivors and their families have shown efficacy in promoting emotional recovery. Group psychotherapy sessions in burn camps appear to positively support emotional responses post-burn, including self-esteem and integration [Figure 3].

## Conclusion

Recently, pediatric burn rehabilitation has been focused on meaningful, practical, sustainable activities that relate to the child’s social life. Burn injuries present significant barriers to community integration, but many children can successfully return to school and other activities. All members of a burn management team interact throughout a child’s recovery and social integration period, from the burn onset to the long term. Pediatric burn rehabilitation is not only about minimizing the effect of physical impairment but also helping the burned children gain independence in the community and improving the QOL of the children and their families.
